# Novel devices for implant-based breast reconstruction: is the use of meshes to support the lower pole justified in terms of benefits? A review of the evidence

**DOI:** 10.3332/ecancer.2018.796

**Published:** 2018-01-10

**Authors:** Lorna Jane Cook, Tibor Kovacs

**Affiliations:** Guy’s and St Thomas’ NHS Trust, London SE11 4TX, UK

**Keywords:** breast reconstruction, implants, acellular dermal matrices, complications and benefits

## Abstract

The use of novel devices such as acellular dermal matrices (ADMs) to support the lower pole in implant-based breast reconstructions (IBBRs) has been described as one of the most important advances in breast reconstructive surgery following mastectomy. However, the majority of outcomes studies focus primarily on providing evidence for the rates of short-term complications associated with their use, as opposed to their reported benefits. Given the high costs associated with using ADMs, together with an increasing number of alternative, cheaper synthetic products entering the market, it is important to clarify whether their use is actually justified and whether the alternative products offer equivalent or superior outcomes. The purpose of this article is to present a comprehensive and updated review of the evidence for the benefits of using different products for lower pole support (LPS) in IBBR compared to reconstructions without. A secondary aim was to determine if there is any evidence to support the use of one product over another.

## Introduction

Increased survival from breast cancer as a result of advances in both diagnosis and treatment has meant that ‘quality-of-life’ measures are becoming an increasingly important indicator of treatment success, as opposed to mortality rates alone [1]. Since breast reconstruction after mastectomy is associated with significant improvements in both psychosocial outcomes and body image, such surgery has become a key consideration in the multidisciplinary management of breast cancer [2]. Consequently, novel techniques and devices in the field of breast reconstruction surgery have been developed with the aim of improving outcomes and meeting patient expectations.

Implant-based breast reconstruction (IBBR) is the most commonly used technique and offers a safe, simple approach to reconstructive surgery, without the need for a long operation or use of donor site tissue [3, 4] Traditionally, a two-stage ‘sub-muscular’ technique has been used, in which, following mastectomy, a ‘pocket’ is developed under the chest wall musculature into which an expander-type implant is placed and the skin flaps are closed over it. The expander implant is then sequentially inflated with saline over a period of weeks until the desired size is reached, after which it is exchanged for a definitive or ‘fixed volume’ implant as a second procedure [2]. The nature of the muscular pocket can either be ‘total’ (in which the pectoralis major, serratus anterior and rectus abdominalis muscle or fascia are elevated and sutured together anterior to the implant); or ‘partial’, where only the upper pole is covered by the pectoralis major [5–7].

Both of these traditional sub-muscular techniques are, however, associated with disadvantages. With partial sub-muscular coverage, the inferior pole is covered only by the skin of the mastectomy flap, predisposing to implant extrusion or exposure, particularly in the presence of necrosis or infection [7, 8]. While total sub-muscular coverage of the implant protects it from potential exposure, it requires extensive muscular dissection and may cause pain during the expansion phase. Both techniques can result in lack of control over the position of the inframammary fold (IMF), in a flat unnatural look or make it difficult to achieve a natural looking ptosis [9, 10].

## The ‘lower pole support’ (LPS) technique

In an attempt to overcome some of these limitations, the traditional sub-muscular technique has been modified in recent years through the additional use of novel materials as adjuncts. Whilst still placing the implant beneath the pectoralis muscle, the ‘LPS’ technique additionally employs the use of a biological or synthetic mesh to cover the lower pole of the implant by suturing it to the IMF and the lower border of the pectoralis major muscle [9, 11]. This technique has the advantage not only of providing support to the lower pole of the implant by acting as a sling but also increases the overall size of the pocket such that, in selected cases, there is the option to proceed directly to a one-stage procedure using a fixed volume implant [12]. When first introduced, the mesh used for this technique was the acellular dermal matrix (ADM) ‘Alloderm’, which is derived from human cadaveric dermis [13, 14]. Since then, multiple alternative products have entered the market – an overview of which is provided below.

## Materials currently used for the ‘LPS’ technique

### Acellular matrices

ADMs are sterile, acellular, biological pieces of material derived from human or animal skin, in which the dermis is stripped of the cellular components, leaving a structurally intact and biochemically inert, extracellular matrix [15]. While the human skin-derived ADM, ‘Alloderm’ was the first ADM to be described in the literature, multiple ADMs derived from both allogenic and xenograft (porcine and bovine) donor sources are now in use. These products differ in their processing and as a result have differences in handling, incorporation, shelf life and cost. In addition to ADMs, de-cellularised tissue derived from other tissue sources, such as the pericardium and peritoneum, have also been developed which have similar properties [16, 17] (Table 1).

### Alternative meshes

The widespread acceptance of the technique together with concerns regarding the high cost of ADMs has led to the development of alternative synthetic mesh types for use in IBBR [18].

### Ti-LOOP Bra

TiLOOP Bra (pfm medical titanium, Nuremberg, Germany) is a non-absorbable, titanium coated polypropylene mesh (TCPM), which has been approved for use in breast reconstruction since 2008. It has a knitted monofilament structure and comes available in three different bra-like sizes. Production involves introducing titanium in gaseous form so that it reaches all parts of the mesh, forming covalent bonds with the plastic surface [19].

### SeriSilk

SERIsilk is a silk-derived biological scaffold in which silk filaments are combined by helical twisting to form a multifilament fibre which is purified and then assembled into a three-dimensional scaffold. Laboratory studies have suggested that it behaves more like an ADM in vivo than a Vicryl mesh, in which it is not just absorbed but accompanied by new tissue generation such that the strength and load bearing properties are transferred to the newly ingrown tissue [20].

### TIGR® Matrix Surgical Mesh

TIGR® Matrix is an absorbable, macroporous mesh knotted from two different degradable fibres – a fast degrading fibre and a slow degrading fibre. The fast degrading fibre is a co-polymer between glycolide and trimethylene carbonate and the slow degrading fibre is a co-polymer between lactide and trimethylene. The idea is that the fast degrading fibre gives extra support during the wound healing phase but is totally resorbed after four months, while the slow absorbing fibre keeps its mechanics up to 6–9 months and is not completely reabsorbed until three years later [21].

### Vicryl mesh

Vicryl mesh is comprised of polyglactin 910 and is cheap, ready to use and widely available. It also exhibits minimal inflammatory reaction, is non-allergenic and resistant to bacteria biofilm formation. One of the greatest benefits of using Vicryl meshes as an alternative to ADMs is the cost difference, estimated at being two-thirds less [22].

### Benefits of using a mesh as an adjunct in IBBR

There are several reported benefits associated with the use of an ADM in IBBR, which include improved cosmesis, better patient satisfaction, less post-operative pain, less capsular contracture and improved cost-effectiveness (mainly as a result of facilitating one-stage procedures and reducing time to completion of fill in two-stage procedures). Some of these benefits are believed to also be associated with the use of the synthetic meshes but with much less associated cost. Whilst there have been multiple literature and systematic reviews exploring outcomes of ADM use in IBBR, their focus has been predominantly on complication profiles and process evaluation rather than evaluating the available evidence for the benefits of their use [23–27].

## Objectives

The purpose of this article is to present a comprehensive and updated review of the literature on the evidence for the benefits of using a mesh in IBBR, compared to reconstructions without a mesh. A secondary aim was to identify any studies which compared the outcomes of using an ADM with an alternative synthetic mesh.

## Methods

### Search strategy

We searched the Ovid SP versions of EMBASE and MEDLINE (last updated 01/7/2017) for relevant articles using the search strategy detailed in Table 2.

### Inclusion criteria

- Studies published from 2007 onwards
- Studies reporting on outcomes following the use of a mesh for LPS in IBBR after mastectomy (to include ADMs, all other biologics and synthetic meshes)
- Comparative studies only either comparing outcomes between mesh and non-mesh reconstructions or between reconstructions using different mesh types
- Clinical outcome studies reporting on at least one of:
  1. cosmetic outcomes (assessed via an objective method);
  2. capsular contracture rates (assessed using the Baker scale)
  3. post-operative pain;
  4. patient-reported outcomes (using a validated method);
  5. cost-effectiveness (based on individual patient data not literature review/theoretical).

### Exclusion criteria

- Single cohort case series without a comparator
- Comparative studies where comparator cohort does not meet the inclusion criteria above
- Non-clinical, animal- or lab-based studies
- Studies reporting solely on cosmetic or revision procedures
- Non-English language articles
- Conference abstracts or abstracts without full text available.

### Data extraction

Data extracted from the included papers were author, journal, year of publication, outcome measure(s) described, study type, participants per cohort compared, mesh types used, methodology used, outcomes per cohort and follow-up periods.

## Results and Discussion

**Figure 1.**
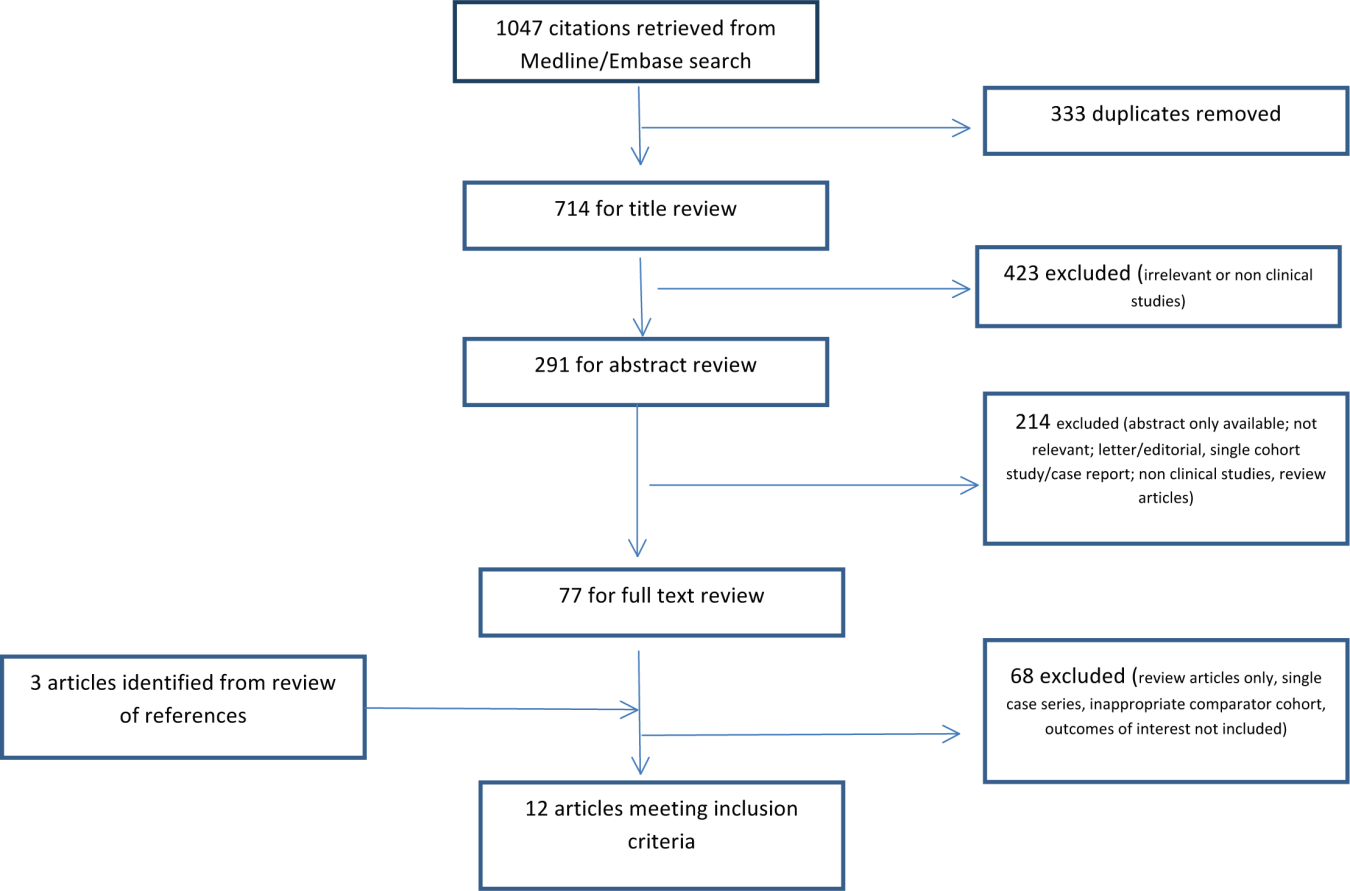
PRISMA diagram.

We identified a total of 12 unique articles that met the inclusion criteria. Of these 10/12 compared ADM-based reconstructions to non-mesh reconstructions, 1/12 compared synthetic mesh (TiLOOP) reconstructions to non-mesh reconstructions and 1/12 compared ADM reconstructions to TiLOOP reconstructions (Figure 1). There were two RCTs with the remainder being non-randomised comparative retrospective cohort studies. A narrative description of the included studies grouped per mesh type and outcome measure reported is given below.

## Evidence for the benefits of using ADMs versus non ADM reconstructions

### Evidence for improved cosmesis

We identified four articles meeting the inclusion criteria which compared the cosmetic outcomes between ADM and non-ADM reconstructions [28–31]. All the four studies used panel assessment of standardised clinical post-operative photographs and a validated scoring system, although the specific methodology differed between studies (Table 3). The ADM type used was specified in two of the studies as being Alloderm [30] and both Alloderm and Surgimend [28], and was unspecified in the remaining two.

All the studies reported that patients in the ADM cohorts had significantly higher overall cosmetic scores compared to the non-ADM cohorts. However, the follow-up period was either not stated per cohort or was significantly different. Given that satisfaction with cosmetic outcomes has been shown to decline over time [32], this may have had a differential effect on the outcome. Furthermore, the studies were not randomised and there were significant differences between the cohorts at baseline in two of the studies (Table 3).

### Evidence for improved Patient-Reported Outcome Measures (PROMs)

Two articles compared PROMs between ADM and non-ADM reconstructions using a validated method (Table 4) [33, 34]. McCathy *et al* [33] randomised patients undergoing two-stage IBBR following mastectomy into receiving Alloderm or not. By using the ‘chest and upper body morbidity’ domains of the BREASTQ questionnaire as well as a visual analogue scale for postoperative pain, they found no significant difference between the two cohorts either immediately post-operatively or during the expansion phase. However, as described in further detail in the following section, there may have been limitations in the study design/methodology that impacted on these results.

The second study by Hanna *et al* [34] used the ‘Breast Evaluation Questionnaire’ to assess whether there was a difference in PROMs between 31 patients who underwent IBBR with Alloderm and 44 patients with total sub-muscular coverage. No significant difference between the two cohorts was demonstrated in all areas of the questionnaire. The only factors identified which influenced the scores were whether reconstructions were bilateral or unilateral and whether patients underwent treatment with radiotherapy or not. In addition to this being a small study, however, the response rate was low overall at 45.3% and there was a difference of over 10 months in length of follow-up between the two cohorts. Furthermore, this was a non-randomised study and there was no stated reason as to why patients were allocated into one cohort or the other.

### Evidence for improved pain outcomes

McCarthy *et al* [33], in addition to using the BREASTQ and visual analogue scale for pain described above, objectively evaluated pain outcomes using 24-hour post-operative narcotic use (reported as oral codeine equivalent). They reported no significant difference between the two cohorts in terms of narcotic requirements (p = 0.38). However, the size of the ADM used (4 × 16 cm) was significantly smaller than that generally used in practice, such that the full effect of increased pocket size on reducing pain, particularly during the expansion phase, may not have been realised as a result (Table 5).

The second study by Seth *et al* [35] was a non-randomised retrospective cohort study which compared outcomes between patients having two-stage reconstruction with and without Alloderm. Patients were additionally stratified according to whether or not the patients received post-operative radiotherapy. Post-operative pain was assessed according to whether it had been specifically documented in patient notes on at least one occasion during the period of follow-up. There was no significant difference in reported pain between the cohorts overall. However, when patients were additionally stratified into those who did or did not have post-mastectomy radiotherapy, the non-ADM cohort reported a significantly higher prevalence of pain in the radiotherapy group, whereas no difference was seen in the ADM cohort. Although the sample size is small, the authors suggest this difference may be a consequence of the protective effect of the ADM in reducing capsular contracture.

### Evidence for reduced levels of capsular contracture

Despite encouraging evidence from single cohort case series which report very low rates of capsular contracture when compared to pre-existing data from non-ADM cohorts [36, 37], only two comparative studies were identified that met the inclusion criteria [29, 30] (Table 6). Vardanian *et al* [30] performed a retrospective comparison of the outcomes of two-stage reconstruction with or without the use of Alloderm ADM in 203 patients and reported a significantly lower rate of capsular contracture (Baker grade III or IV) in the ADM cohort at a mean follow-up of 29 months (3.8% versus 19.4% p < 0.001). A second study by Forsberg *et al* [29] also found capsular contracture was significantly lower, although the ADM type used was not specified (8.1% versus 23.5% p = 0.048).

Both of these comparative studies have potential sources of bias, however, which limit how far their results can be accepted. Both the studies were retrospective and based on case note review alone as opposed to prospective and standardised evaluation of Baker grade of contracture. Furthermore, capsular contracture rate is known to increase over time and both the studies had short median follow-up periods, which was significantly less in the ADM cohort in the study of Forsberg *et al* [29] and was not reported on a per cohort basis in the study of Vardanian *et al* [30].

### Evidence for cost–benefits

Three non-randomised comparative cohort studies addressed the question of a cost–benefit when comparing ADM reconstructions with non-ADM reconstructions. Three additional articles were identified but were excluded as they used literature searches/systematic reviews to pool data on outcomes from case series rather than using patient level data [38–40]. All three included studies used different methodologies (Table 7).

Two studies evaluated the cost of using Strattice in a single-stage procedure. Johnson *et al* [41] compared the costs of both unilateral and bilateral reconstructions with two-stage tissue expander/implant (TE/I) reconstructions without ADM or one-stage latissimus dorsi (LD) flap + implant reconstructions. They used NHS tariffs as a proxy for actual costs incurred together with the acquisition costs for the Strattice mesh. They reported that for unilateral reconstructions, there was a cost advantage of using Strattice compared to the other two techniques. Specifically, when compared to two-stage TE/I reconstructions, a one-stage Strattice reconstruction eliminated costs associated with outpatient visits for expansions and a second procedure for implant exchange. When compared to LD + implant reconstructions, the lack of additional donor site morbidity and outpatient visits for seroma drainage also resulted in a cost advantage.

Kilchenmann *et al* [42] alternatively used ‘resource allocation’ rather than direct costs to compare unilateral one-stage reconstructions with Strattice to two-stage TE/I reconstructions; two-stage LD-TE/I reconstructions and one-stage LD flap + implant reconstruction. In agreement with Johnson *et al* [41], they also demonstrated a cost advantage to the single-stage approach which offsets the additional cost of the mesh. However, when compared to the one-stage LD + implant reconstructions, they found the costs were equivalent.

Bank *et al* [43] used the number of expansions required to achieve final expander fill volume as a proxy for the number of outpatient clinics attended post-operatively by patients undergoing uncomplicated ADM- and non-ADM two-stage TE/I reconstructions, in order to determine whether there was a difference in direct hospital costs. Although they found ADM reconstructions had indeed reduced the number of outpatient visits and associated costs, this was insufficient to offset the elevated material costs of using an ADM.

Without a standardised means of performing a cost analysis, it is difficult to be certain from these three studies as to whether use of an ADM results in a cost advantage over other techniques. Furthermore, longer term follow-up including details of revision surgery and quality-of-life data is required in order to perform a full assessment.

## Evidence for the benefits of using synthetic meshes versus non-mesh reconstructions

We identified no comparative studies reporting on the outcomes of interest for SeriSilk, TIGR matrix or Vicryl meshes in IBBR.

One retrospective, non-randomised cohort study reported on the difference in outcomes between reconstructions with or without the use of a TiLOOP bra mesh (Table 8). Dieterich *et al* [44] compared 42 patients with a TiLOOP mesh to 42 who underwent a non-mesh reconstruction, using the BREASTQ post-reconstruction questionnaire. Analysis of responses showed no significant differences between the groups in all of the domains of the BREASTQ. However, stepwise linear regression showed a negative association of the ‘satisfaction with breasts’ domain with the use of the TiLOOP bra. Reasons for this difference may have been multifactorial, however. The way in which patients were selected into the TiLOOP cohort depended on intra-operative findings such as adequacy of soft tissue coverage; comparison of the two groups at baseline showed that the patients in the TiLOOP cohort had significantly lower BMIs and were significantly younger – both factors which have an impact on expectations of aesthetic outcome.

Capsular contracture rates were additionally compared between the two cohorts with 7/42 in the non-TiLOOP group and 2/42 in the TiLOOP, developing a Baker III/IV contracture during the period of follow-up (p = 0.052). Cosmesis, post-operative pain and costs were not compared between the two cohorts.

## Studies comparing outcomes of reconstructions using ADMs versus synthetic meshes

A single study was identified which compared outcomes (complications, cosmesis and PROMs) between synthetic mesh and ADM reconstructions [45]. Gschwantler *et al* [45] performed a prospective, randomised multicentre pilot study in which patients undergoing immediate breast reconstruction were allocated to receiving TiLOOP or a porcine ADM (Protexa). Patients in the ADM cohort differed significantly from the TiLOOP cohort at baseline in terms of exposure to radiotherapy and also in terms of rates of reconstructive failure (protexa n = 7 30.4%; TiLOOP n = 2 7.7%).

Cosmetic outcomes were again scored using standardised post-operative photographs at six months using the four point Harris scale, by four surgeons and two external experts. They reported that cosmetic scores were significantly higher in the TiLOOP cohort. However, the ADM cohort had a significantly higher proportion of reconstructive failures, and when these cases were removed from the analysis there was no longer any significant differences found in terms of cosmesis.

Patient-reported outcomes were assessed using the EORTC QLQ C30 and BR23 questionnaires. Significantly lower scores were reported in the ADM cohort at the first post-operative visit for arm pain (p = 0.039) and fatigue (p = 0.03) and at six months for effect on family life (p = 0.021) and sexual interest (p = 0.039)

Given the small numbers of patients compared, and the unusually high rate of reconstructive failure in the ADM cohort, it is difficult to draw any clear conclusions from this study.

## Conclusions

Despite the popularity of using meshes as adjuncts to support the lower pole in implant-based reconstruction, this review has demonstrated a significant paucity of evidence to support the reported benefits of their use. Where there is evidence available, this is primarily for the use of ADMs rather than the newer synthetic meshes and is mainly derived from retrospective cohort studies. The need to demonstrate equivalent or superior outcomes with the use of synthetic meshes compared to ADMs is an important consideration, given that they are much less expensive to produce. Furthermore, without high-quality comparative data it is impossible for surgeons to know which mesh they should use in order to give the greatest benefit to their patients.

Previous literature reviews have focused on the complication and safety profiles of the meshes used in breast reconstruction as the primary outcome measures of interest [46–50]. Given that the primary purpose of breast reconstruction surgery is to improve psychosocial functioning and body image, it is perhaps equally important that outcomes such as cosmesis and patient satisfaction are also included as a means of evaluating the success of the technique [51]. One of the problems with this, however, is that there is as yet no agreed method by which cosmesis, PROMs and cost-benefit should be assessed objectively. Current methods used to evaluate cosmesis, as reported in this review, which involve a panel review of post-operative photographs are associated with a degree of subjectivity and inter-assessor variability [52].

The majority of the evidence base for use of meshes in implant-based reconstruction is composed of either single-cohort case series or retrospective cohort studies [53]. Ideally, well-designed prospective cohort studies or RCTs with sufficient periods of follow-up should be carried out to determine whether the cost of these products is justified in terms of the benefits provided. The iBRA study (implant-based Breast Reconstruction evaluAtion) is a UK multicentre audit which is designed to explore the practice and outcomes of implant-based reconstructions [54]. Data derived from this study will not only provide a large amount of prospective data but will help to inform the design of any future trials in this area.

## Figures and Tables

**Table 1.** ADMs and synthetic meshes commonly used in IBBR.

Category	Product name	Source/material	Details
**ADM**	Alloderm	Human dermis	Freeze-dried, aseptic, requires refrigeration and rehydration prior to use
	Alloderm RTU		Sterile, pre-hydrated, two-minute soak only
	Alloderm contour fenestrated		Sterile, pre-hydrated, crescent shape to ease use and fenestrated to allow passage of any periprosthetic fluid
	FlexHD		Pre-hydrated
	DermaMatrix		Freeze-dried aseptic, requires rehydration
	CGDerm		Freeze dried, requires 20 minutes’ rehydration
	CGCryoDerm		Frozen but not dried, only required three minutes’ soak in saline
	Strattice	Porcine dermis	Sterile
	Protexa	Porcine dermis	Sterile
	Surgimend	Bovine dermis	Sterile
**Other biologics**	Veritas Meso BioMatrix	Bovine pericardium Porcine preitoneum	Fenestrated mesh made of decellularised pericardium Derived from porcine mesothelium
**Synthetic mesh**	TiLOOP bra	Titanised polypropelene	Non-absorbable mesh
	TIGR mesh	Absorbable mesh	Macropourous mesh made of two types of co-polymer fibres
	Vicryl Mesh	Absorbable mesh	Dissolves rather than integrates into tissue
**Other materials**	SERI silk	Multifilament silk mesh	Behaves like an ADM *in vivo* allowing in-growth of new tissue

**Table 2.** Search strategy.

No	Term
1	Breast/
2	Breast.mp
3	1 or 2
4	Biocompatible materials/
5	ADM.mp
6	Acellular derm* .mp
7	strattice.mp
8	Surgimend.mp
9	Dermamatrix.mp
10	Alloderm.mp
11	Allomax.mp
12	FlexHD.mp
13	TIGR mesh.mp
14	TiLOOP.mp
15	Veritas.mp
16	Seri.mp
17	4 or 5 or 6 or 7 or 8 or 9 or 10 or 11 or 12 or 13 or 14 or 15 or 16
18	Reconstructive surgical procedures/
19	‘Prostheses and implants’/
20	Breast implants/
21	Tissue expansion devices/
22	Implant*.mp
23	Expand*.mp
24	Prosthe*.mp
25	Surgery, plastic/
26	18 or 19 or 20 or 21 or 22 or 23 or 24 or 25
27	3 and 17 and 26
28	Limit 27 to Humans, English Language
29	Remove duplicates

**Table 3.** Cosmetic outcome studies – ADM versus non-ADM cohort.

Author	Cohorts compared	Variables compared at baseline	Assessors/method used	Follow-up period/time of assessment	Method of evaluation	Results
**Ibrahim *et al* [28], 2015**	ADM (Alloderm or Surgimend) n = 18 patients Non-ADM n = 20 patients	Yes NSD	Panel assessment of pre-/post-operative photographs by five plastic surgeons not directly involved in care and blinded	Six months to seven years (1.7 years) Not compared between groups	Scored using validated subscales for volume; contour; placement of implant; scars; lower pole projection; IMF definition	ADM cohort scored statistically significantly higher in terms of overall cosmetic outcome and for subscales for contour and implant placement
**Forsberg *et al* [29], 2014**	ADM (type not specified) n = 58 reconstructions Non-ADM n = 125 reconstructions	Yes – significantly more delayed reconstructions in non-ADM cohort	Panel assessment of post-operative photographs; 18 blinded assessors (six plastic surgeons; six trainees; six medical students)	ADM: 25 months Non-ADM: 34 months Significant difference p = 0.005	Scored using validated subscales for contour; symmetry of shape; symmetry of size; position and overall outcome	ADM cohort received higher scores for all parameters in each group of assessors. Difference reached significance for majority
**Nguyen *et al* [31], 2012**	ADM (type not specified) n = 53 patients Non-ADM n = 58	Yes – significantly higher BMI in ADM cohort	Panel assessment of post-operative photographs; three plastic surgeons not involved in care; blinded	Not specified, although photographs taken at least 90 days following second-stage procedure	Scored using validated subscales for volume; contour; placement of implant; scars; lower pole projection; IMF definition	ADM cohort scored statistically significantly higher in terms of overall cosmetic outcome and for subscales for volume, IMF definition and implant placement
**Vardanian *et al* [30], 2011**	ADM (Alloderm) n = 208 reconstructions Non-ADM – partial (n = 119) and total (n = 10) sub-muscular	Yes NSD	Panel assessment of post-operative photographs; four blinded assessors – surgeon, secretary and two medical students	Not stated – all post-implant exchange	Four-point Harris scale for overall aesthetic outcome and IMF placement (1 – poor; 2 – fair; 3 – good; 4 – excellent)	Score for both overall aesthetic outcome and IMF placement significantly higher in ADM cohort

**NSD: no significant difference.**

**Table 4.** Studies comparing PROMs between ADM versus non-ADM reconstructions.

Reference	Methods/materials compared	Selection into cohort	Validated PROMS instrument used/subscales	Response rate	Follow-up period	Results	Comments
McCarthy *et al* [33], 2012	ADM: Alloderm n = 36 patients Non-ADM: n = 33 patients	Randomised	The Physical Well-being: chest and upper body domain of the BREASTQ pre-/post-reconstruction module Visual analogue scale Prospective	100%	Immediately post-operatively; following the 1st–3rd post-op expansions and immediately prior to implant exchange	No significant difference in scores for either BREASTQ or VAS at all stages of reconstruction measured	Recruitment stopped early due to slow accrual. Smaller sized mesh used than is generally used in current practice
Hanna *et al* [34], 2013	ADM: Alloderm n = 31 patients Non-ADM n = 44	Method not stated, consecutive patients	Breast Evaluation Questionnaire (phone assessment), retrospective	45.3% Rate between groups not compared	ADM 10.2 +/- 7.7 m TSR 20.7 +/- 11 m No p value stated	No significant difference between the two cohorts in terms of responses to all questions.	Low response rate and small patient population

**Table 5.** Studies comparing pain outcomes between cohorts.

Reference	Methods/materials compared	Selection into cohort	Method of pain assessment	Results	Follow-up period (timing of assessment)	Conclusions
McCarthy *et al* [33], 2012	ADM: Alloderm n = 36 patients Non-ADM: n = 33 patients	Randomised	PROMS (see Table 2) 24-hour post-operative narcotic use (reported as oral codeine equivalent)	No significant difference in scores for either BREASTQ or VAS at all stages of reconstruction measured. No significant difference in post-operative narcotic requirements p = 0.38	Immediately post-operatively, following the 1st–3rd post-op expansions and immediately prior to implant exchange. 24 hours post-operatively	No evidence for reduction in post-operative pain with use of an ADM either immediately or in the expansion phase of two-stage breast reconstruction
Seth *et al* [35], 2012	ADM: Alloderm/Flex HD N = 199 breasts/137 patients No ADM 393 breasts/280 patients	Not stated	Recorded if documented by surgeon following at least one subjective patient complaint	ADM: Pain documented for five patients (2.5%) and for ten non-ADM patients (2.5%) p = 0.60. No difference in the ADM cohort when stratified according to radiotherapy exposure; significantly more patients reported pain if exposed to radiotherapy in the non-ADM cohort	ADM 23.2 +/− 8.9 months (3–45) Non ADM24.4 +/− 12.7 (4–49) p = 0.23	Authors suggest that this differential effect of radiotherapy on pain may be a consequence of the protective effect of the ADM due to reduced capsular contracture

**Table 6.** Comparative studies evaluating capsular contracture (cc) rates (ADM versus Non-ADM).

Reference	Groups compared	Variables	Method of assessment	Follow-up period	Results
Vardanian *et al* [30], 2011	ADM (Alloderm) n = 208 Non-ADM – partial (n = 119) and total (n = 10) sub-muscular	No significant difference (NSD) between cohorts in terms of age, BMI, smoking and indication for reconstruction	Retrospective chart review of recorded Baker score. Considered significant if Baker III or IV	Median 29 months post-implant exchange for both cohorts – not compared. Range not reported	Overall cc rates: ADM 3.8%; non-ADM 19.4% p < 0.001. On multivariate analysis ADM use associated with significantly lower cc rates (OR 0.18; 95% CI 0.08–0.43)
Forsberg *et al* [29], 2014	ADM (type not specified) n = 58 Non-ADM n = 125	NSD between cohorts in terms of age, BMI, smoking, diabetes, adjuvant therapy, implant type (saline/silicone) or size. Significant difference in the number of immediate reconstructions (higher in ADM cohort) and length of follow-up period	Retrospective chart review of recorded Baker score. Considered significant if Baker III or IV	ADM 24.6 months Non-ADM 33.8 months p = 0.005	Significant difference in cc rates: ADM: 8.1% Non-ADM 23.5% P = 0.048

**Table 7.** Studies comparing cost-effectiveness (ADM versus Non-ADM).

Reference	Study type	Reconstructions compared	Method	Factors included	Results
Johnson *et al* [41], 2013	Single-centre cohort, retrospective	Strattice: bilateral (n = 13), unilateral (n = 11) TE/I: bilateral n = 12, unilateral n = 10 LD + implant n = 10	Use of National Tariffs (NHS England 2011–2012) as a proxy for hospital costs plus acquisition costs for Strattice	Cost of index operation, consumables in addition to those accounted for in-tariff payment, admissions and attendances and complications	For unilateral cases, Strattice is less costlier than TE/I (£3685 vs. £4985) and LD-based reconstructions (£3685 vs. £6321) For bilateral cases, Strattice is costlier than TE/I due to anomaly in reimbursement system where bilateral mastectomy does not attract any higher reimbursement than unilateral
Kilchenmann *et al* [42], 2014	Single-centre cohort, retrospective	Unilateral reconstructions only Strattice one-stage n = 25 TE/I n = 27 LD+ implant n = 32 LD/TE n = 17	Use of resources utilised rather than costs incurred	Cost of initial operation, additional hospitalisations and operative procedures; outpatient appointments, seroma aspiration, complication rates	Unilateral single-stage ADM reconstructions were associated with fewer resources utilised compared to TE/I and LD/TE/I in both complicated and non-complicated cases over a 24-m period. LD + mplantI and ADM cohorts equivalent
Bank *et al* [43], 2013	Single-centre, retrospective	Uncomplicated reconstructions only TE/I with ADM (Strattice or Alloderm) *n* = 84 Non-ADM TE/I *n* = 48	Number of tissue expansions required to meet final fill volume as a proxy for number of outpatient clinic encounters	Total cost of each clinic encounter using expenditure data from centre (faculty fees, labour fees, material costs). Cost of ADM	Although fewer clinic visits are required to achieve final fill volume in ADM cohort the savings made did not offset the cost of using an ADM. Difference of $3000 remained if Alloderm is used and $2500 if Strattice

**Table 8.** Study comparing PROMS between synthetic mesh (TiLOOP) and non-mesh reconstruction.

Reference	Methods/materials compared	Selection into cohort	Validated PROMS instrument used/subscales	Response rate	Follow-up period	Results	Comments
Dieterich *et al* [44], 2015	TiLOOP bra n = 42 Non-mesh – n = 42 Retrospective cohort	Specific selection into TiLOOP cohort was based on decision made intra-operatively	BREASTQ – post-reconstruction module (all subscales) – postal questionnaire, retrospective	67.7% NSD between two groups p = 0.117	TiLOOP 18 m (1–40) No mesh 17.5 m (1–83) P = 0.827	No significant differences between the groups in all of the domains. However, stepwise linear regression showed a negative association with “satisfaction with breasts” scores in the TiLOOP cohort	Surgeon selection into cohort and significant differences between two groups in terms of BMI and age
